# Efficacy of electroacupuncture with different frequencies in the treatment of chemotherapy-induced peripheral neuropathy: A study protocol for a randomized controlled trial

**DOI:** 10.3389/fneur.2022.843886

**Published:** 2022-07-28

**Authors:** Chao Lu, Wenlong Bao, Dehou Deng, Rongrong Li, Guangliang Li, Shanlin Zou, Yan Wang

**Affiliations:** ^1^The Traditional Chinese Medicine Department, The Cancer Hospital of the University of Chinese Academy of Sciences (Zhejiang Cancer Hospital), Hangzhou, China; ^2^The Third Clinical Medical College, Zhejiang University of Traditional Chinese Medicine, Hangzhou, China; ^3^Breast Medical Oncology Department, The Cancer Hospital of the University of Chinese Academy of Sciences (Zhejiang Cancer Hospital), Hangzhou, China

**Keywords:** electroacupuncture, chemotherapy-induced peripheral neuropathy, randomized controlled trial, protocol, Mecobalamin

## Abstract

**Introduction:**

Chemotherapy-induced peripheral neuropathy (CIPN) is a common complication in patients with cancer during chemotherapy. It mainly leads to severe numbness of the hands and feet and causes great pain in patients. Electroacupuncture (EA) is considered to be beneficial in improving peripheral neuropathy and relieving numbness of the hands and feet. This trial aims to evaluate the therapeutic effect of different frequencies of EA on CIPN in patients with cancer.

**Methods and analysis:**

This study is a randomized controlled trial. In total, 160 eligible CIPN patients are randomly assigned to the 2 Hz EA group, 100 Hz EA group, 2/100 Hz EA group, and control group in the ratio of 1:1:1:1. All patients in the EA treatment groups receive treatment with EA three times a week for 4 weeks and following up for 4 weeks. The patients in the control group are given Mecobalamin (MeCbl) tablets orally, one tablet at a time, three times a day, for 4 weeks, and following up for 4 weeks. The primary outcome measures are the participant neurotoxicity questionnaire (PNQ) and the peripheral neurotoxicity assessment rating (NCI CTCAE V5.0). Secondary outcomes are the quality of life scale (EORTC QLQ-C30) and the measurement of peripheral nerve conduction velocity (NCV). The results are evaluated at baseline, post-treatment phase, and following up for 4 weeks. All major analyses are based on the intention to treat principle.

**Ethics/dissemination:**

This protocol was approved by the Medical Ethics Committee of the Cancer Hospital of the University of Chinese Academy of Sciences (Zhejiang Cancer Hospital) on 7 December 2021. The license number is IRB-2021-458. This study provides clinical efficacy data of different frequencies of EA in the treatment of CIPN. The results help to prove whether EA is an effective therapy for CIPN and optimize the frequency of EA for CIPN. The results of this study are shared with health care professionals, the public, and relevant organizations through the publication of manuscripts and conference reports.

**Trial registration number:**

ChiCTR2100054458.

## Background

Cancer is a common disease endangering human health. Chemotherapy is still one of the main treatment methods, but chemotherapy drugs often cause different side effects while killing cancer cells. Chemotherapy-induced peripheral neuropathy (CIPN) is a common side effect of many chemotherapeutic drugs, especially platinum compounds, vinblastine, taxanes, and other chemotherapeutic drugs. For example, oxaliplatin is the third generation of broad-spectrum platinum compounds anticancer drugs after cisplatin and carboplatin. It is widely used in the treatment of gastrointestinal malignancies (1). However, the primary adverse reaction of oxaliplatin is CIPN, and the incidence of acute neurotoxicity can be as high as 85−95% (2). In contrast, its chronic neurotoxicity is closely related to the cumulative dose of drugs. It was reported that when the drug dose reaches 1,170 mg/m^2^, the incidence of neurotoxicity above grade III can be as high as 50% (3). About 40% of clinical colorectal cancer patients reduce the dose or even stop oxaliplatin due to CIPN; thus, the expected therapeutic effect cannot be achieved (4).

The main symptoms of CIPN are peripheral nerve damage, limb numbness, loss of sensation or abnormal sensation (such as tingling, electric shock and sensation, foreign body sensation, etc.), decreased tendon reflex, and sensory ataxia, which can be triggered or aggravated in case of cold stimulation. Based on the severity of symptoms, it is often divided into four levels. However, its pathogenesis is not clear. It is mainly considered to be related to the toxic accumulation of oxaliplatin itself and the metabolite oxalate. The main target of oxaliplatin is the dorsal root ganglia (DRG) without blood-brain barrier protection. The accumulation rate of chemotherapeutic drugs in DRG neurons is much higher than the clearance rate. This accumulation inhibits the rRNA synthesis in neurons, impairs the protein synthesis, and leads to the damage and degeneration of neurons (5). In addition, the measurement of nerve conduction velocity (NCV) is an important index for diagnosing and evaluating peripheral neuropathy and an objective standard for evaluating the degree of peripheral nerve damage (6). For example, peripheral neuropathy caused by diabetes can make the peripheral NCV slow. By measuring NCV, the degree of peripheral nerve damage can be assessed (7). CIPN caused by chemotherapeutic drugs can also significantly reduce the conduction velocity of peripheral nerves (8–11). According to current guidelines, there is no curative therapy while duloxetine is the only medication proven to be effective in alleviating the symptoms of CIPN. Antiepileptic drugs, calcium magnesium mixture, antioxidants, neuroprotective agents, and nutrients are generally used to treat CIPN, but they often bring other toxic and side effects or unsatisfactory curative effects. CIPN caused by chemotherapy drugs affect the treatment process of cancer and easily cause emotional instability and seriously affect the quality of life. Therefore, there is an urgent need to find an effective and less toxic and side effects treatment for CIPN.

Acupuncture, an important part of traditional Chinese medicine, played a prominent role in treating cancer-related diseases, especially in preventing and treating complications caused by chemotherapeutic drugs (11, 12). In addition, some studies have shown that acupuncture can effectively treat kinds of peripheral neuropathy, improve peripheral NCV, and alleviate patients' sensory abnormalities or pain symptoms at the end of limbs (13). Electroacupuncture (EA) is a new technology of acupuncture. It was developed by combining traditional acupuncture technology with electrical stimulation. In recent years, it has been widely used in clinical treatment. Many animal experiments show that EA can effectively treat CIPN, and the analgesic effect of low-frequency EA is better. For example, in the CIPN animal model, low-frequency EA (2Hz) can reduce the mechanical pain and hyperalgesia of CIPN induced by paclitaxel in rats (14) and also has a pronounced therapeutic effect on CIPN caused by oxaliplatin in rats (15); low frequency EA (10 Hz) is more effective than high frequency EA (100 Hz) in alleviating paclitaxel induced CIPN symptoms (16). While, in clinical research, it was reported that high-frequency EA (50–100 Hz) could achieve a good curative effect in the treatment of CIPN caused by oxaliplatin (17); Many researchers have also used density waves 2/100 Hz (18) and 2/120 Hz (19) to treat peripheral neuropathy and achieved positive results. However, some studies have pointed out that the efficacy of EA on CIPN is not clear. It is not as good as simply using drugs (20) and even aggravates the peripheral nerve symptoms of patients (21). According to the above, we can evaluate the degree of CIPN damage and treatment recovery by measuring NCV, and the clinical efficacy of EA in the treatment of CIPN needs to be further verified; The clinical effectiveness of different frequencies of EA in the treatment of CIPN may be different. Therefore, we designed an RCT involving drug control to accurately evaluate the efficacy of EA on CIPN and optimize the selection of EA frequency.

## Methods

### Objective

To demonstrate whether EA is an effective therapy for CIPN, the curative effect of EA with different frequencies on CIPN and its effect on NCV is observed.

### Hypotheses

1. EA can significantly improve CIPN symptoms compared with drug intervention, as subjective (participant neurotoxicity questionnaire, PNQ) and objective (peripheral nerve conduction velocity, NCV) measurements after treatment.
2. EA can comprehensively improve patients' quality of life while improving CIPN, as measured by the quality of life scale (EORTC QLQ-C30).
3. There are differences in the efficacy of different frequencies of EA on CIPN.

### Trial design

**Figure 1:**
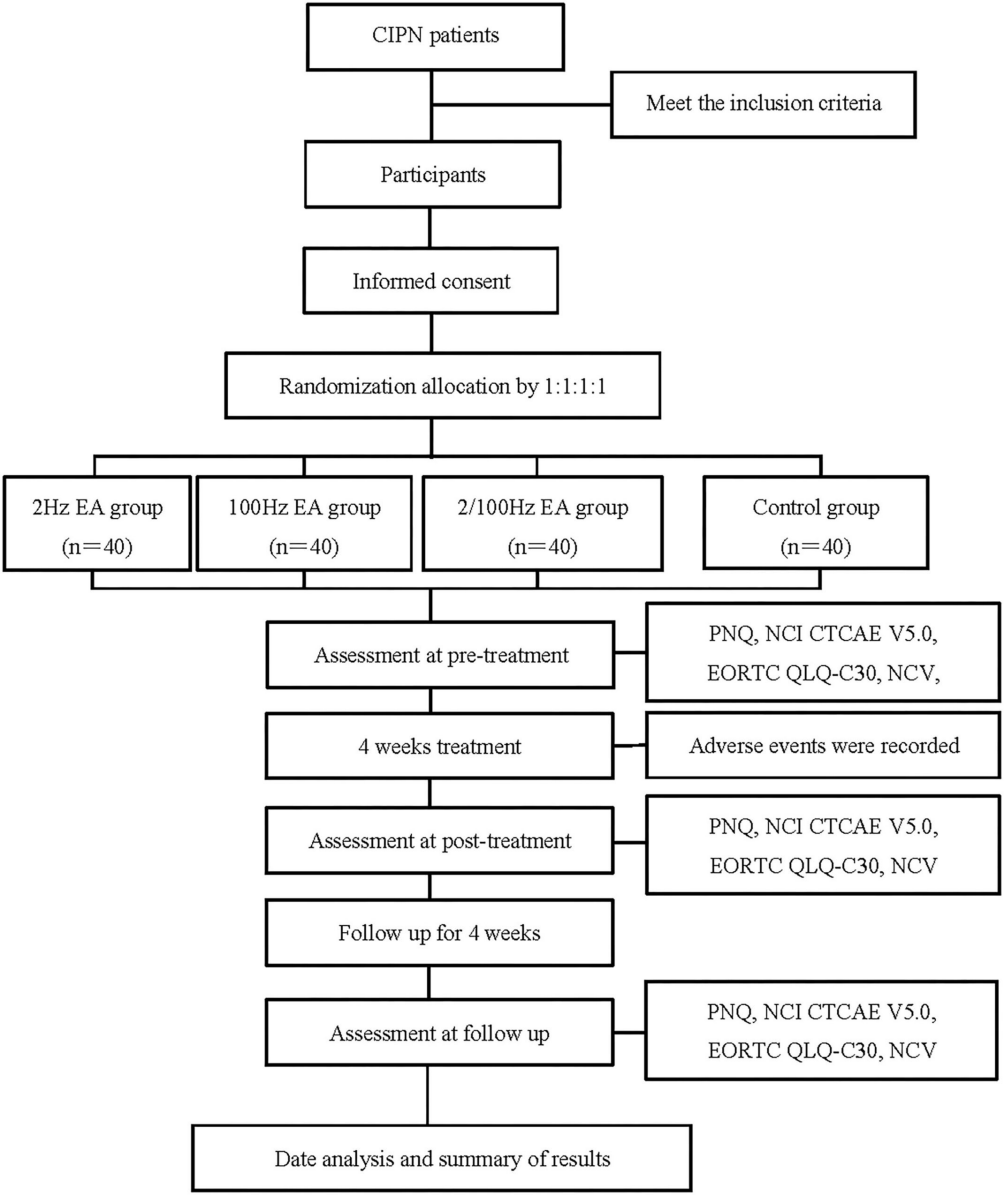
Flow chart of the study process. CIPN, Chemotherapy-induced peripheral neuropathy; EA, Electroacupuncture; PNQ, The Participant Neurotoxicity Questionnaire; NCI CTCAE V5.0, National Cancer Institute Common Terminology Criteria for Adverse Events Version 5.0; EORTC QLQ-C30, The European O-rganization for Reasearch and Treatment of Cancer, Quality of Life Questionnare-Core 30; NCV, Nerve Conduction Velocity.

This trial was designed as a randomized, controlled, participant, researcher, and outcome evaluator blind experimental study to compare the therapeutic effects of three different frequencies of EA regimens on CIPN patients. In total, 160 participants with CIPN were randomly assigned to four treatment groups in the ratio of 1:1:1:1, namely the 2 Hz EA group, 100 Hz EA group, 2/100 Hz EA group, and control group. The flow chart of the study process is shown in Figure 1. Additionally, the trial schedule of enrolment, treatments, and assessments is shown in Table 1. The reporting of this protocol is based on the Standards for Reporting Interventions in Clinical Trials of Acupuncture (STRICTA) (22) and the SPIRIT reporting guidelines (23).

**Table 1:** Schedule of enrolment, treatments, and assessments.

**Study period**	**Screening**	**Baseline Week 0**	**Treatment period Week 4**	**Follow-up period Week 8**
Eligibility screening	**○**			
Demographic data	**○**			
Case data	**○**			
Inclusion criteria	**○**			
Exclusion criteria	**○**			
Informed consent	**○**			
**Treatment**		**○**	**○**	
**Outcome assessment**				
(1) PNQ		**○**	**○**	**○**
(2) NCI CTCAE V5.0		**○**	**○**	**○**
(3) EORTC QLQ-C30		**○**	**○**	**○**
(4) NCV		**○**	**○**	**○**
Safety assessment		**○**	**○**	

***○**, required; PNQ, The Participant Neurotoxicity Questionnaire; NCI CTCAE V5.0, National Cancer Institute Common Terminology Criteria for Adverse Events Version 5.0; EORTC QLQ-C30, The European O-rganization for Reasearch and Treatment of Cancer, Quality of Life Questionnare-Core 30; NCV, Nerve Conduction Velocity*.

### Participants and recruitment

Participants with CIPN were recruited at the Cancer Hospital of the University of Chinese Academy of Sciences (Zhejiang Cancer Hospital). Recruitment posters were placed in the outpatient hall to increase exposure, and the recruitment information for this trial was informed through online publicity.

### Inclusion criteria

Participants who met all of the following requirements were considered for inclusion: (1) Over 18 and under 70 years old, and the expected survival time was ≥1 year; (2) Pathological diagnosis was a malignant tumor, and peripheral neurotoxicity above grade 1 occurred after chemotherapy with platinum compounds, paclitaxel, and vinblastine; (3) Have daily living ability, no hearing loss, and can cooperate independently to complete all examinations; (4) No severe heart, liver, brain, kidney and other diseases, Karnofsky functional status score (KPS) >60; (5) Clear consciousness without mental illness or cognitive impairment; (6) Signed the written informed consent form for the clinical trial.

### Exclusion criteria

Patients were excluded if they were (1) diagnosed with relatively severe systemic diseases (cardiovascular disease, acute infectious disease, hematopathy, et al.); (2) diagnosed with severe mental disorders, like schizophrenia; (3) diagnosed with peripheral neuropathy caused by other causes, such as peripheral nerve injury or diabetic peripheral neuropathy; (4) pregnant and lactating women; (5) alcohol and/or other drug abuse or dependent; (6) participating in other clinical trials of acupuncture or drugs.

### Randomization and allocation concealment

The randomization was performed by the Research Center of the Scientific Department, the Cancer Hospital of the University of Chinese Academy of Sciences (Zhejiang Cancer Hospital). A randomization method was used to generate the random allocation sequence of four groups; four groups of random serial numbers were randomly placed in opaque sealed envelopes by other staff to ensure the concealment of distribution. The opaque sealed envelope was printed with the screening sequence of participants on the outside and randomly assigned groups on the inside, consecutively numbered and connected into a series. According to the subject's visit order, the envelope was selected with the corresponding serial number.

### Blinding and informed consent

This is a participant-assessor-blinded trial in which participants are not aware of their group assignments. Participants were informed that they have a 25% chance of being allocated to receive either of the four treatments: 2 Hz EA group, 100 Hz EA group, 2/100 Hz EA group, and drugs in the control group. The follow-up assessors were also unaware of the patient's group allocation. Although acupuncturists were not blinded to the treatment assignments, they were not involved in the outcome assessments or data analyses.

To achieve blinding, the kind of disposable sterile steel needles, selection of acupoints, treatment times, and skin disinfection process were formed consistently and significantly. Meanwhile, the only variable was the frequency of EA. Participants were blinded to their group allocation.

## Intervention

### Intervention group

(1) 2 Hz EA group: acupuncture needle was routinely used. Table 2 shows the location of all selected acupoints. Bilateral Hegu (LI4), Houxi (SI3), Waiguan (SJ5), Quchi (LI11), and Baxie (EX-UE9) acupoints were taken in the upper limbs. Bilateral Zusanli (ST36), Yinlingquan (GB34), Yanglingquan (SP9), Sanyinjiao (SP6), Taichong (LR3), and Bafeng (EX-LE10) acupoints were taken in the lower limbs; Upper limb EA connects Waiguan (SJ5) and Hegu (LI4) points; Lower limb EA connects Zusanli (ST36) and Sanyinjiao(SP6) points. The frequency of 2 Hz was used in this group, and the stimulation intensity was based on the patient's tolerance. Each treatment time was 30 min, three times a week for 4 weeks. (2) 100 Hz EA group: the treatment method was the same as that of the 2 Hz EA group, while the EA frequency was 100 Hz. (3) 2/100 Hz EA group: the treatment method was the same as that of the 2 Hz EA group, and the EA frequency was 2/100 Hz. (Equipment: the disposable acupuncture needle adopts Huatuo brand filiform needle produced by Suzhou instrument factory, with models of 0.25 ^*^ 40 MM. The electro acupuncture adopts Hans-200a model).

**Table 2:** Location of acupoints for treating CIPN.

**Acupoints**	**Location**
Hegu (LI4, bilateral)	On the back of the hand, between the first and second metacarpals, at the midpoint of the radial side of the second metacarpal
Houxi (SI3, bilateral)	Slightly clench the fist, the distal side of the ulnar side behind the palmar joint of the fifth finger, the horizontal pattern of the palm, and the red and white flesh border of the head
Waiguan (SJ5, bilateral)	Two cun above the transverse crease of the dorsal wrist, on the line connecting Yangchi (SJ4) and Zhoujian (EX-UE1), the midpoint of the gap between ulna and radius
Quchi (LI11, bilateral)	Bend the elbow at a right angle when the elbow bends at the end of the transverse line
Baxie (EX-UE9, bilateral)	There are 8 points on the back of the hand, behind the edge of the web between the first and fifth fingers, between the red and white flesh, left and right
Zusanli (ST36, bilateral)	Three cun below Dubi (ST35), one finger -breadth (middle finger) from the anterior crest of tibia
Yanglingquan (GB34, bilateral)	On the outside of the lower leg, when the fibular head is in the depression before and below
Yinlingquan (SP9, bilateral)	In the depression between the medial lower edge of the lower tibia and the medial edge of the tibia
Sanyinjiao (SP6, bilateral)	Three cun above the medial malleolus, on the posterior border of the medial aspect of tibia
Taichong (LR3, bilateral)	In the dorsum of the foot, between the first and second metatarsals, and in the depression in front of the metatarsal junction
Bafeng (EX-LE10, bilateral)	Between the first to fifth toes, at the red and white flesh border behind the toe web edge, there are four acupoints on one side, with a total of eight acupoints on the left and right

### Control group

The participants were given Mecobalamin (MeCbl) tablets orally, one tablet each time, three times a day for 4 weeks.

### Outcome measures

The participant neurotoxicity questionnaire (PNQ) and peripheral neurotoxicity were assessed according to NCI CTCAE version 5.0, EORTC quality of life scale (QLQ-C30), and NCV measurement was the outcomes used to assess efficacy. Adverse events were recorded to assess safety.

#### Primary outcome

##### The participant neurotoxicity questionnaire

The participant neurotoxicity questionnaire is a questionnaire for diagnosis and quantification of CIPN (24, 25), which is validated and rated the highest overall assessment (26, 27). It includes two items (sensory nerve and motor nerve) and mainly assesses symptoms of paresthesia such as numbness, pain, tingling, and cold perception in the hands or feet. It was chosen as the primary assessment of neuropathy symptoms based on its capacity to detect both sensory nerve deficit and interference of neuropathy symptoms in activities of daily living (24, 28), as well as provide a robust patient-level assessment of neuropathy symptoms (26). The PNQ grades symptoms from grade 0 (no neuropathy) to grade 4 (very severe neuropathy).

##### Peripheral neurotoxicity assessment according to NCI CTCAE version 5.0

A total of five grades of peripheral neurotoxicity were identified according to NCI CTCAE version5.0 (29). Grade I, tendon reflex disappeared or sensory numbness (including acupuncture sensation), but does not affect function; Grade II, sensory loss or sensory numbness (including acupuncture sensation), which affects the function but does not affect the activities of daily life; grade III, sensory loss or sensory numbness (including acupuncture sensation), which affects the activities of daily life; grade IV, sensory motor neuropathy, which significantly interferes with the activities of daily life.

#### Secondary outcome

##### European Organization for research and treatment of Cancer quality of life scale (QLQ-C30)

The European Organization for research and treatment of Cancer (EORTC) QLQ-C30 is a quality-of-life instrument for use in international clinical trials in oncology (30). We used it to evaluate patients' quality of life before treatment, after treatment, and follow-up. QLQ-C30 scale includes five functional subscales (body, function, cognition, emotion, and Society), three symptom subscales (fatigue, pain, and nausea and vomiting), six single symptom measurement items (dyspnea, loss of appetite, insomnia, constipation, diarrhea, and economic difficulties), and one overall health subscale. The higher the score of the functional subscale, the better the quality of life. The higher the score of the symptom subscale, the worse the quality of life.

##### NCV measurement

Nerve conduction velocity detects the speed of nerve response by placing electrodes on the skin and stimulating nerves with electrical pulses. The room temperature was kept at 20–25°C during the examination. Before and after treatment, the motor conduction velocity (MCV) of the median nerve of the upper limb and the common peroneal nerve of the lower limb, and the sensory conduction velocity (SCV) of the median nerve and sural nerve were measured. The median nerve was stimulated at the wrist and elbow and recorded in the thenar and adductor pollicis muscles. The common peroneal nerve was stimulated at the ankle, under the fibular head and popliteal fossa, and the recording point was the extensor digitorum brevis. The sural nerve was stimulated at the middle and lower 1/3 junction of the lower leg, recorded at the lateral malleolus. The side with apparent feeling was selected for detection in both limbs. If both sides feel the same, one side was selected.

#### Other outcomes

All kinds of adverse reactions during treatment were recorded, including specific manifestations, occurrence time, treatment measures, results, and follow-up and indicated whether the case was continued to be included. In addition, all vital signs and adverse reactions were measured and recorded at each visit.

#### Setting

The trial was conducted in the Cancer Hospital of the University of Chinese Academy of Sciences (Zhejiang Cancer Hospital) between 1 January 2022 and 31 December 2024. This protocol was approved by the Medical Ethical Committee of the Cancer Hospital of the University of Chinese Academy of Sciences (Zhejiang Cancer Hospital) on 7 December 2021. The license number is IRB-2021-458.

The eligibility of prospective participants was determined by a researcher who was not involved in the assessment or treatment of the participants. On the first arrival for the screening meeting, patients were given more detailed information about the study procedures. Before the first treatment and after the last treatment, all patients were required to complete the PNQ, NCI CTCAE V5.0, EORTC QLQ-C30, and NCV measurement. After 4 weeks of treatment, follow-up was carried out, and the above indicators were measured again to evaluate the curative effect. The participants cannot take pain medications or unspecified CIPN-related therapeutic drugs during treatment. If the patient had to take pain medications or drugs that interfere with the results of the research due to changes in his condition, the patient would be considered to drop out. In order to promote enrollment and participant compliance, all treatment costs and all outcome measurements were free for participants.

## Statistical methods

### Sample size

This is a prospective clinical study. According to the randomized control scheme, three treatment groups and one control group are designed, with 40 patients in each group.

### Statistical analysis

De-identified outcome data were analyzed by a statistician blinded to group allocations using the Statistical Package for the Social Sciences (SPSS) V 24.0 statistical software package. The data analysis was based on the intention-to-treat (ITT) population. All statistical analyses were two-sided tests except for the primary outcome. The level of significance was established at 0.05. Continuous data were represented by the mean, standard deviation, median, minimum value, and maximum value; categorical data were represented by percentages. To compare two independent samples, a *t*-test or nonparametric test was used for continuous data, and a chi-square test/the Fisher exact test/nonparametric tests were used for categorical data. For safety analysis, the incidence of adverse events was compared between the four groups using the chi-square test or the Fisher exact test.

### Patient safety and quality control

All acupuncturists and evaluators were required to undergo special training before the trial to ensure consistent practices. The training program included CIPN diagnosis, participant inclusion, and exclusion criteria, location of the acupoints, EA techniques, and evaluation criteria of case report forms. Dropouts and withdrawals from the study were recorded through the intervention periods.

Any adverse events (described as unfavorable or unintended signs, symptoms, or diseases) related to acupuncture treatment were observed and reported by patients and practitioners during each patient visit. In addition, all vital signs and adverse events were measured and recorded at each visit. Throughout the trial, all study data were collected in the case report forms and entered into the electronic data capture system by independent researchers.

This trial is monitored by the scientific research department of the Cancer Hospital of the University of Chinese Academy of Sciences (Zhejiang Cancer Hospital). They are responsible for monitoring the data and have the right to reveal blinded data and can verify the authenticity between the raw data and the recorded data to guarantee accuracy and quality throughout the study.

## Discussion

Chemotherapy-induced peripheral neuropathy is one of the most frequent side effects caused by antineoplastic agents, with a prevalence from 19 to over 85% (31). Clinically, CIPN is a primarily sensory neuropathy that may be accompanied by motor and autonomic changes of varying intensity and duration (32). Due to its high prevalence, CIPN constitutes a significant clinical problem for patients with cancer and their health care providers, mainly because there is no single effective method to prevent CIPN. According to previously published data, we suggest that EA therapy may be effective in CIPN, while the dominant frequency of EA in the treatment of CIPN is not clear. Thus, we used EA technology as the primary treatment method and oral drugs as the control. Different frequencies of EA were used to treat CIPN for the first time. The curative effects of 2, 100, and 2/100 Hz EA were compared horizontally.

Selecting the appropriate acupuncture points to treat CIPN better and achieve the expected curative effect is one of the critical problems that need to be solved. We have screened the current high-frequency acupoints of acupuncture in the treatment of CIPN through published data (33, 34) and consulted experienced acupuncturists to determine the combination selection of acupoints.

Currently, there are no definite criteria or tools for evaluating CIPN (32, 35). Thus, selecting the suitable efficacy evaluation index to objectively evaluate the clinical efficacy of EA in the treatment of CIPN is also a critical problem that needs to be solved. In this study, the severity of CIPN was evaluated using the internationally recognized PNQ as the subjective indicator. Researchers graded the severity of CIPN according to the PNQ and clinical symptoms concerning NCI CTCAE version 5.0 and measured NCV as objective indicators to evaluate the degree of peripheral nerve injury and clinical treatment effect of CIPN patients. The quality of life of patients was comprehensively evaluated according to EORTC QLQ-C30, and adverse events during the study were recorded, which made the study results more convincing.

Mecobalamin is an active form of vitamin B12 that has been suggested to be beneficial in improving nerve conduction and neuropathic pain symptoms. As an auxiliary agent, it exerts neuronal protection by promoting regeneration of injured nerves and antagonizing glutamate-induced neurotoxicity and is already widely used to treat peripheral neuropathies (36, 37). Some clinical trials have shown that MeCbl can improve the numbness of hands and feet caused by peripheral neuropathy (38, 39). MeCbl is considered a safe, effective, and generally well-tolerated drug for treating peripheral neuropathies (36). Therefore, we used MeCbl as the treatment scheme for the control group to clarify the efficacy of EA.

By comparing the clinical efficacy of 2, 100, and 2/100Hz EA in the treatment of CIPN, this study can further clarify the effect of EA in the treatment of CIPN, optimize the selection of EA frequency, and provide technical guidance for clinical treatment. The results will further provide new clues, new ideas, and new methods for acupuncture in the treatment of nervous system diseases and cancer-related diseases. Finally, we hope the results of this study will effectively improve the CIPN symptoms of patients with tumors, reduce patients' pain, and improve their quality of life.

## Ethics statement

The studies involving human participants were reviewed and approved by the Medical Ethics Committee of the Cancer Hospital of the University of Chinese Academy of Sciences (Zhejiang Cancer Hospital), on December 7, 2021. The license number is IRB-2021-458. The patients/participants provided their written informed consent to participate in this study.

## Author contributions

CL and RL contributed to the study's design, drafting, and editing of the manuscript. CL wrote the first manuscript for this trial and edited the final manuscript. RL revised the manuscript. DD participated in the design of the trial. GL evaluated participant information and collected CRF data. WB and SZ conducted the acupuncture operation. YW registered the study result data. All authors read and approved the final manuscript.

## Funding

The work was supported by the Program of Science Research Foundation of Zhejiang Provincial TCM Administration (2022ZB058).

## Conflict of interest

The authors declare that the research was conducted in the absence of any commercial or financial relationships that could be construed as a potential conflict of interest.

## Publisher's note

All claims expressed in this article are solely those of the authors and do not necessarily represent those of their affiliated organizations, or those of the publisher, the editors and the reviewers. Any product that may be evaluated in this article, or claim that may be made by its manufacturer, is not guaranteed or endorsed by the publisher.
